# Rho Kinases in Embryonic Development and Stem Cell Research

**DOI:** 10.1007/s00005-022-00642-z

**Published:** 2022-01-19

**Authors:** Jianjian Shi, Lei Wei

**Affiliations:** Herman B Wells Center for Pediatric Research, Department of Pediatrics, School of Medicine, Indiana University, 1044 West Walnut Street, R4-370, Indianapolis, IN 46202-5225 USA

**Keywords:** ROCK, Isoform, Inhibitor, Development, Stem cell

## Abstract

The Rho-associated coiled-coil containing kinases (ROCKs or Rho kinases) belong to the AGC (PKA/PKG/PKC) family of serine/threonine kinases and are major downstream effectors of small GTPase RhoA, a key regulator of actin-cytoskeleton reorganization. The ROCK family contains two members, ROCK1 and ROCK2, which share 65% overall identity and 92% identity in kinase domain. ROCK1 and ROCK2 were assumed to be functionally redundant, based largely on their major common activators, their high degree kinase domain homology, and study results from overexpression with kinase constructs or chemical inhibitors. ROCK signaling research has expanded to all areas of biology and medicine since its discovery in 1996. The rapid advance is befitting ROCK’s versatile functions in modulating various cell behavior, such as contraction, adhesion, migration, proliferation, polarity, cytokinesis, and differentiation. The rapid advance is noticeably driven by an extensive linking with clinical medicine, including cardiovascular abnormalities, aberrant immune responsive, and cancer development and metastasis. The rapid advance during the past decade is further powered by novel biotechnologies including CRISPR-Cas and single cell omics. Current consensus, derived mainly from gene targeting and RNA interference approaches, is that the two ROCK isoforms have overlapping and distinct cellular, physiological and pathophysiology roles. In this review, we present an overview of the milestone discoveries in ROCK research. We then focus on the current understanding of ROCK signaling in embryonic development, current research status using knockout and knockin mouse models, and stem cell research.

## Introduction

Rho kinases (Rho-associated coiled-coil-containing protein kinase, hereafter referred to as ROCKs) are major downstream effectors of the small GTPase RhoA (Ishizaki et al. 1996; Leung et al. 1996; Matsui et al. 1996; Nakagawa et al. 1996). The ROCK family contains two members, ROCK1 (also called ROKβ or p160ROCK) and ROCK2 (also known as ROKα), which share 65% overall identity in amino acid sequence and 92% identity in their kinase domains. Both kinases contain a catalytic kinase domain at the N-terminus, followed by a central coiled-coil domain including a Rho-binding domain (RBD), and a carboxyl-terminal pleckstrin-homology (PH) domain with an internal cysteine-rich domain. In humans and mice, both ROCK1 and ROCK2 are ubiquitously expressed across tissues (Nakagawa et al. 1996).

Over the past 25 years, the ROCK family has attracted significant attention as a promising therapeutic target for a wide spectra of human diseases, including cardiovascular diseases, pulmonary diseases, neurodegenerative diseases, metabolic disorders, ocular diseases, and cancers (Budzyn et al. 2006; Chrissobolis and Sobey 2006; Dai et al. 2018; Feng et al. 2016; Huang et al. 2015; Landry et al. 2020; Narumiya and Thumkeo 2018; Saadeldin et al. 2020; Shi and Wei 2007, 2013; Shimokawa 2020; Surma et al. 2011; Wei et al. 2016; Yu et al. 2020). Due to the high degree of amino acid sequence homology, some of the biological functions of ROCK1 and ROCK2 are therefore believed overlapping and compensatory, including actin cytoskeleton organization, smooth muscle cell contraction, cell proliferation, adhesion, migration, polarity, cytokinesis, differentiation and survival in many cell types. However, they are not completely compensatory as over-activation of ROCK1 or ROCK2 in a disease state can cause pathological consequence (Surma et al. 2011; Wong et al. 2009). Two relatively selective ROCK inhibitors, Y27632 (Uehata et al. 1997) and fasudil (Asano et al. 1989), have been widely used to dissect the roles of ROCK in cellular signaling. In animal disease models, a large body of data has supported that inhibition of ROCK has a promising application in disease therapy. Both Y27632 and fasudil bind to the kinase domain and inhibit ROCK1 and ROCK2 with similar potency (Breitenlechner et al. 2003; Davies et al. 2000; Ishizaki et al. 2000; Uehata et al. 1997). Due to their tremendous therapeutic potential, a significant number of ROCK inhibitors have been developed (Defert and Boland 2017; Feng and LoGrasso 2014; Feng et al. 2016). Nonetheless, fasudil remains the only systemic ROCK inhibitor used in humans for cerebral vasospasm after surgery of subarachnoid hemorrhage in Japan (Shibuya et al. 1992) and has been tested in numerous clinical trials in other countries, with the majority of trials focusing on cardiovascular diseases (Fukumoto et al. 2013; Shi and Wei 2013; Shibuya et al. 2005; Surma et al. 2011; Vicari et al. 2005).

Different from the well-established shared functions of ROCK isoforms, the distinct roles of ROCK1 and ROCK2 are still not well understood (Dai et al. 2018; Feng et al. 2016; Hartmann et al. 2015; Landry et al. 2020; Narumiya and Thumkeo 2018; Saadeldin et al. 2020; Shahbazi et al. 2020; Shi and Wei 2007, 2013; Shi et al. 2011; Shimokawa 2020; Surma et al. 2011; Wei et al. 2016; Yu et al. 2020; Zanin-Zhorov et al. 2016; Zhang et al. 2006). The specific disruption of each ROCK isoform by gene targeting in mice, using short interfering RNA (siRNA)-based gene silencing in cells and CRISPR gene editing provides growing evidence of distinct cellular, physiological and pathophysiological functions of the two isoforms. Introduction of KD025, the first highly selective ROCK2-isoform inhibitor (Boerma et al. 2008), allows novel exploration of its therapeutic potential in various vascular, immune and neuronal disorders (Akhter et al. 2018; Boerma et al. 2008; Flynn et al. 2016; Lee et al. 2014; Sadeghian et al. 2018; Sharma and Roy 2020; Weiss et al. 2016; Zanin-Zhorov et al. 2014, 2016, 2017). In this review, we will summarize the milestone discoveries in ROCK research and the current understanding of ROCK signaling in embryonic development, update current discoveries from knockout and knockin mouse models, and stem cell research.

## Milestone Discoveries in ROCK Research

**Fig. 1:**
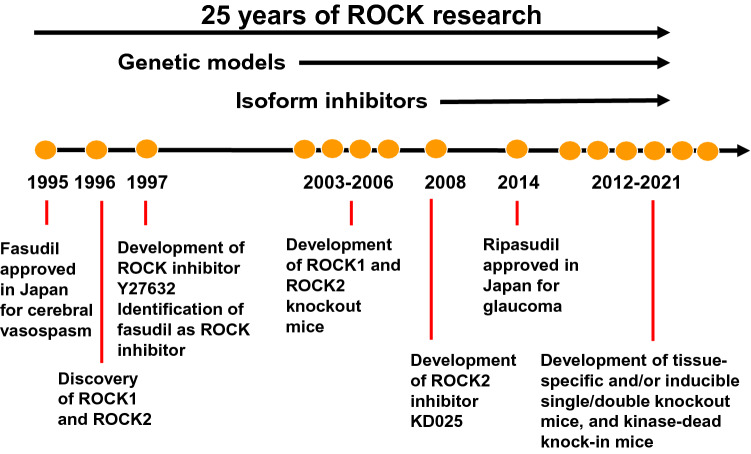
Milestone discoveries in ROCK research

Protein kinases play a vital regulatory role in nearly every aspect of cell biology through modifying protein phosphorylation status to influence numerous cell functions. The origin of protein kinase research traces back to the discovery of ATP-dependent, divalent metal ion-dependent enzymatic activity in the mid-1950s by Fischer and Krebs (1955), which ultimately led to the discovery of the serine/threonine kinase phosphorylase b kinase. The discovery of the Rho GTPase family can be traced back to the mid-1980s when Rho was identified as key molecule for actin reorganization, from which Rho signaling research has been expanded to all areas of biology and medicine (Narumiya and Thumkeo 2018). In the mid-1990s when ROCKs were recognized as the major effectors in Rho-induced actin reorganization, it was marked as a pivotal point in Rho signaling research (Fig. 1).

### Discovery of ROCKs

In 1996, ROCK1 and ROCK2 were discovered by three independent groups and originally called ROKβ/p160ROCK and ROKα, respectively (Ishizaki et al. 1996; Leung et al. 1996; Matsui et al. 1996; Nakagawa et al. 1996). The ROCK-mediated signaling pathway was then identified in smooth muscle cells and connected to cardiovascular diseases with abnormal smooth muscle contraction (Amano et al. 1996; Kawano et al. 1999; Kimura et al. 1996; Kureishi et al. 1997). Both isoforms were reported to phosphorylate the same major downstream substrates such as the myosin binding subunit of myosin light chain phosphatase (MYPT1) (Amano et al. 1996; Kawano et al. 1999; Kimura et al. 1996), myosin light chain (MLC) (Amano et al. 1996; Kureishi et al. 1997), LIM-kinases (LIMK) (Maekawa et al. 1999; Ohashi et al. 2000; Sumi et al. 2001), thereby modulating actin cytoskeleton organization, stress fiber formation and cell contraction. By far, this signaling pathway remains the best characterized mechanisms for interpreting the roles of ROCKs in regulating actin cytoskeleton in many cell types: ROCKs promote actomyosin contractility through increasing MLC phosphorylation, and stabilize actin filaments through LIMK activation, resulting in cofilin phosphorylation and thereby inhibiting its actin-depolymerization activity.

These early ROCK studies support the paradigm that both ROCK isoforms are functionally redundant due to the high degree of amino acid sequence homology in their kinase domains, shared activators (Rho GTPases and lipid mediators) and substrates, and auto-inhibitory activity (Amano et al. 1999). In their inactive form, the carboxyl terminal PH domain and RBD of ROCK interact with the kinase domain, which forms an auto-inhibitory loop. The RBD localized in the coiled-coil domain interacts only with activated Rho GTPases including RhoA, RhoB and RhoC (Fujisawa et al. 1996). The PH domain is believed to interact with lipid mediators such as arachidonic acid and sphingosylphosphorylcholine (Feng et al. 1999; Fu et al. 1998; Shirao et al. 2002). In addition to the canonical substrates (MYPT1, MLC and LIMK), ROCKs share more than 30 immediate downstream substrates and novel ROCK substrates are constantly being discovered (reviewed in: Amano et al. 2010; Shi and Wei 2007; Surma et al. 2011; Wei et al. 2016).

### Development and Therapeutic Effects of ROCK Inhibitors

The efforts in drug discovery targeting on ROCKs were concentrated on the development of non-isoform selective ROCK inhibitors in the early period based on the assumption of functionally redundant isoforms. The most commonly used chemical inhibitors are fasudil (also named HA-1077) and Y27632 (Uehata et al. 1997). Fasudil was originally discovered for inhibiting PKA and PKC (Asano et al. 1989), but was later identified to be more potent for inhibiting ROCKs (Davies et al. 2000; Uehata et al. 1997). Fasudil is also the only ROCK inhibitor used in humans systemically for the prevention and treatment of cerebral vasospasm after surgery in subarachnoid hemorrhagic patients in Japan (Shibuya et al. 1992). Hydroxyfasudil is the main metabolite of fasudil after oral administration, and H-1152P is another analogue of fasudil, both of which are more potent than fasudil. Because these inhibitors target the ATP-dependent kinase domain of ROCK1 and ROCK2, they are non-isoform specific and also able to inhibit other serine/threonine kinases such as PKA and PKC at higher dosages (Bain et al. 2007; Davies et al. 2000).

ROCK inhibitors were initially investigated for therapeutic potential in vascular diseases, such as cerebral vasospasm, hypertension and coronary artery spasm (Sasaki et al. 2002; Shibuya et al. 2005; Suzuki et al. 2007; Uehata et al. 1997; Zhao et al. 2006), and gradually extended to metabolic, neurodegenerative and inflammatory diseases, etc. (Fukumoto and Shimokawa 2013; Huang et al. 2013; Knipe et al. 2015; Sawada and Liao 2014; Shi and Wei 2013; Watzlawick et al. 2014). Moreover, ROCK inhibition has been extended to cancer treatment during the recent decade (Morgan-Fisher et al. 2013; Shahbazi et al. 2020; Wei et al. 2016). Due to general promising results of ROCK pan-inhibition and increasing evidence challenging the old paradigm, considerable interest and efforts are devoted to the development of more potent and isoform-selective ROCK inhibitors (Defert and Boland 2017; Feng and LoGrasso 2014; Feng et al. 2016). Among these novel ROCK inhibitors, ripasudil (also named K-115), a close analogue of fasudil, was approved in Japan in 2014 for the treatment of glaucoma (Garnock-Jones 2014). It should be noted, inhibition of ROCKs can relax smooth muscle cells, ROCK pan-inhibitors potentially trigger a rapid and pronounced drop in blood pressure upon systemic exposure (Defert and Boland 2017; Feng and LoGrasso 2014; Feng et al. 2016). In addition, a reversible decrease in lymphocyte counts is another potential adverse effect that may be caused by systemic exposure of ROCK pan-inhibitors (Shaw et al. 2014). Therefore, the unwanted systemic side effects of ROCK pan-inhibitors have so far hampered their clinical applications.

Unfortunately, due to the 65% similarly in overall amino acid sequence and 92% similarity in kinase domains, developing isoform-selective inhibitors is incredibly challenging. Therefore, isoform-selective inhibition of ROCKs without major side effects is considered a significant breakthrough for systemic applications. Notably, KD025 (also named SLx-2119) was published in 2008, and it is the first highly selective ROCK2-isoform inhibitor achieving a high isoform selectivity of > 200-fold for ROCK2 vs. ROCK1 (Boerma et al. 2008). Its therapeutic potential has been explored in fibrotic disease (Boerma et al. 2008), focal cerebral ischemia (Akhter et al. 2018; Lee et al. 2014; Sadeghian et al. 2018), and auto-immune disease (Flynn et al. 2016; Weiss et al. 2016; Zanin-Zhorov and Blazar 2021; Zanin-Zhorov et al. 2014, 2016, 2017). Interestingly, hypotensive phenotype was not observed when KD025 was tested in systemic application (Lee et al. 2014). In contrast to the recent progress in the development of ROCK2-selective inhibitors, ROCK1 inhibitors with > 50-fold selectivity for ROCK1 vs. ROCK2 have not been reported although they are highly desired (Defert and Boland 2017; Feng and LoGrasso 2014; Feng et al. 2016).

### ROCK Isoform Functions

In addition to the shared activators and substrates described above, a growing body of evidence confirms that both ROCK isoforms have distinct partners that interact with each other in individual cell types, which in turn can perform non-redundant functions. ROCK1 is cleaved by caspase 3 at the cleavage site DETD1113 during apoptosis and this sequence for caspase 3 cleavage is conserved in human, rat and mouse, but is not present in ROCK2 (Coleman et al. 2001; Sebbagh et al. 2001). On the other hand, during cytotoxic lymphocyte granule-induced cell death, human ROCK2 can be cleaved by the proapoptotic protease granzyme B at IGLD1131 site, but this site is not present in ROCK1 (Sebbagh et al. 2005). Moreover, microRNAs (miRNAs) were found to be involved in regulating gene expression around the time of the discovery of ROCKs (Lee et al. 1993; Wightman et al. 1993). Decades of research has identified numerous miRNAs that participate in regulating ROCK1 and ROCK2 expression and activity in cancer and normal cells (extensively reviewed in two recent articles: Uray et al. 2020; Wei et al. 2016). A miRNA is a small non-coding RNA that guides molecules modulating gene expression primarily by binding to the 3′ untranslated regions (UTR) of targeted messenger RNA, leading to mRNA degradation and decreased translational efficiency (Nilsen 2007). However, translation upregulation by miRNAs has been observed, depending on the target RNA sequence context, associated microribonucleoproteins and cellular conditions (Vasudevan 2012). Since the 3′ UTRs of ROCK1 and ROCK2 are comprised of different sets of miRNA-binding sites, their expressions can be regulated differently by miRNAs. For instance, ROCK1 was found to be a target of miR-143/145 in smooth muscle cells (Xin et al. 2009; Zhang et al. 2016), miR-124, miR-135a, miR-145, miR146a, miR-148a, miR-186, miR-340, miR-584, and miR-1280 in cancer cells (Wei et al. 2016). On the other hand, ROCK2 was found to be a target of miR-34/449 in multiciliated cells (Chevalier et al. 2015; Mercey et al. 2016), miR-142-3p in lymphocytes (Liu et al. 2014), and miR-101, miR-124, miR-138, miR-139, and miR-200b/c in cancer cells (Wei et al. 2016). It is worth noting that some miRNAs target both ROCK1 and ROCK2, such as miR-124 and miR135a in cancer cells (An et al. 2013; Kroiss et al. 2015; Zheng et al. 2012).

The non-redundant function of each isoforms was emerged in recent years, which significantly improve the application of ROCK inhibition in medicine. The specific disruption of each ROCK isoform by gene targeting in mice, siRNA interference in cells and CRISPR gene editing provides more solid evidence revealing distinct cellular, physiological and pathophysiological functions of the two isoforms which can even oppose one another in specialized contexts. In non-tumor and tumor cells, ROCK1 and ROCK2 have been reported to have functional differences in regulating the actin cytoskeleton, adhesion, migration, proliferation and gene expression, but the underlying mechanisms are not fully understood (reviewed in: Amano et al. 2010; Shi and Wei 2007; Surma et al. 2011; Wei et al. 2016). Their functional differences can be explained by their variations in expression level, subcellular location, and interaction partners in diverse cell types (Amano et al. 2010; Shi and Wei 2007; Wei et al. 2016; Surma et al. 2011). For instance, a study of mouse tissues with ROCK isoform-specific antibodies revealed that ROCK1 and ROCK2 have differential distribution and subcellular localization patterns in epithelial, muscle and neural tissues (Iizuka et al. 2012). Our observations of ROCK1- or ROCK2-deficient mouse embryonic fibroblasts (MEFs) suggest that ROCK1 plays a critical role in mediating stress-induced cell injury and death, and ROCK2 plays a pro-survival role in cell injury (Shi et al. 2013a, b; Surma et al. 2014; Wei et al. 2015). In MEFs, only ROCK1 deficiency inhibits doxorubicin-induced disruption of central stress fibers and formation of cortical contractile rings leading to reduced cell detachment (Shi et al. 2013a, b; Surma et al. 2014; Wei et al. 2015). The anti-detachment effects of ROCK1 deficiency responding to this cytotoxic drug is mediated through reduced MLC phosphorylation besides preserved cofilin phosphorylation which lead to the reduced actomyosin contraction and preserved actin polymerization. It is interesting to note that the pro-survival characteristics of ROCK1 deficiency in MEFs is contrary to the anti-growth and pro-apoptotic characteristics of ROCK1 deficiency in oncogene-bearing leukemic cells although it is also associated with reduced MLC phosphorylation (Mali et al. 2011). The differences in functional outcomes of ROCK1 deficiency in MEFs vs. leukemic cells are likely due to variations in anchorage-dependency, cytoskeleton organization and interacting partners in normal vs. tumor cells.

The concept of ROCK isoform-specific functions in pathophysiology is further supported by the studies in animal models with systemic or cell type-specific knockout mice. Global homozygous *ROCK1*^–/–^ (Zhang et al. 2006) and heterozygous *ROCK1*^+/–^ (Rikitake et al. 2005) mice show beneficial effects such as decreased cardiac fibrosis without affecting pressure overload or angiotensin II-induced cardiac hypertrophy. In a gain-of-function mouse model, transgenic mice expressing constitutively active ROCK1 in cardiomyocytes develop fibrotic cardiomyopathy (Yang et al. 2012). These studies support that ROCK1 is a key molecule in mediating apoptotic signaling in cardiomyocytes under pressure overload and in genetically induced cardiomyopathy (Chang et al. 2006; Shi et al. 2008, 2010, 2011; Yue et al. 2014; Zhang et al. 2006). In addition, global ROCK1 deletion or cardiomyocyte-specific ROCK1 deletion restores autophagic flux through reducing Beclin1 phosphorylation in doxorubicin cardiotoxicity (Shi et al. 2018). Collectively, these studies have provided strong evidence that ROCK1 is a vital player for pathologic cardiac fibrosis formation, cardiomyocyte apoptosis and autophagy, but not for hypertrophy. On the other hand, global hemizygous ROCK2 deficient and cardiomyocyte-specific ROCK2-deficient mice were found to be resistant to pressure overload-induced cardiac hypertrophy and fibrosis formation, supporting that ROCK2 is important in mediating the cardiac hypertrophic response (Okamoto et al. 2013; Sunamura et al. 2018). However, there are also inconsistencies among the beneficial versus detrimental effects of single ROCK isoform knockouts due to the excessive compensation resulted from overactivation of the remaining isoform. A recent study reported that cardiomyocyte-specific ROCK1 deficiency worsened pressure overload-induced cardiac dysfunction that is associated with compensatory up-regulation of ROCK2 (Sunamura et al. 2018). On the other hand cardiomyocyte-specific ROCK2 knockout caused compensatory ROCK1 overactivation resulting in increased cardiac fibrosis (Shi et al. 2019). To overcome the restraint from excessive compensation of remained isoform in the single ROCK isoform knockout models, we recently used cell type-specific inducible approach to delete both ROCK isoforms to investigate the short- and long-term effects in the absence of total ROCK activity in cardiomyocytes (Shi et al. 2019). It revealed that ROCKs are not required for maintaining sarcomeric cytoskeleton in adult cardiomyocytes, instead, they participate in the regulation of non-sarcomeric actin cytoskeleton organization, inhibit autophagy by promoting mammalian target of rapamycin activity and contribute to age-related cardiac fibrosis (Shi et al. 2019).

In addition to cardiac pathophysiology, studies using global homozygous *ROCK1*^–/–^ mouse models supported multiple roles of ROCK1 in regulating both normal and abnormal hematopoiesis in different hematopoietic lineages through both actin-based and non-actin based downstream substrates, including maintaining the activation of tumor-suppressor genes (Gallo et al. 2012; Kapur et al. 2016; Mali et al. 2011, 2014; Vemula et al. 2010, 2012; Wen et al. 2012). Particularly, ROCK1 plays either negative roles in regulating inflammatory and erythropoietic stress (Vemula et al. 2010, 2012), or positive roles in regulating the growth and survival of leukemic cells (Mali et al. 2011), and growth and maturation of mast cells (Kapur et al. 2016). In this regard, the role of ROCK2 in regulating both normal and abnormal hematopoiesis as well as downstream substrates in different hematopoietic lineages remains unanswered. The combination of in-depth analyses of cell type-specific double vs. single ROCK knockout mouse models are expected to provide valuable insights to the shared and distinct ROCK isoform functions in pathophysiology.

## ROCKs Play Essential Roles in Various Embryonic Developmental Stages

Along with quickly growing research interest, many dissecting tools became available, comprising of chemical inhibitors, expression vectors, siRNA, transgenic mice, systemic and cell type-specific knockout animals. Consequently, ROCK research has widely covered almost all biological systems and extensively involved in human diseases including cardiovascular diseases, pulmonary diseases, neurodegenerative diseases, metabolic disorders, ocular diseases, and cancers, etc. which are fascinating subjects of many recent reviews (Dai et al. 2018; Feng et al. 2016; Landry et al. 2020; Narumiya and Thumkeo 2018; Saadeldin et al. 2020; Shahbazi et al. 2020; Shi and Wei 2007, 2013; Shimokawa 2020; Surma et al. 2011; Wei et al. 2016; Yu et al. 2020). Here, we focus on the current status of ROCK research in development.

### Overview

RhoA has important roles in many developmental processes and most of our knowledge of RhoA signaling function in mammalian development is from studies in cell type-specific RhoA knockout mice (Duquette and Lamarche-Vane 2014; Narumiya and Thumkeo 2018; Pedersen and Brakebusch 2012; Zhou and Zheng 2013). ROCK1 and ROCK2 are major RhoA downstream effectors, mediating RhoA action on actomyosin bundle formation during development (Narumiya and Thumkeo 2018). Both ROCK1 and ROCK2 are ubiquitously expressed in mouse embryos at every developmental stages (Duan et al. 2014; Kawagishi et al. 2004; Laeno et al. 2013; Saadeldin et al. 2020; Shimizu et al. 2005; Thumkeo et al. 2003; Wei et al. 2001). However, they have evidently distinct preferential expression patterns. We observed that ROCK1 is highly enriched in developing hearts and ROCK2 is ubiquitously expressed at stages E7.5–9.5 (Wei et al. 2001). In *ROCK1*^–/–^ embryos which contain a knockin *lacZ* reporter gene, LacZ staining was detected in many locations throughout the embryo (E13.5–15.5), including the skin, heart, aorta, umbilical blood vessels, and dorsal root ganglia (Shimizu et al. 2005). In *ROCK2*^*–/–*^ embryos with a knockin *lacZ* reporter gene, LacZ staining was also observed in many locations throughout the embryo (E13.5), including the heart, liver, umbilical blood vessels, dorsal root ganglions, and the labyrinth layer of the placenta (Thumkeo et al. 2003).

Numerous studies, using ROCK knockout mice, or transgenic mice of tissue-specific expressing of ROCK dominant negative mutants (inhibiting kinase activity of both isoforms), or mouse and chick treated with ROCK pan-inhibitors (Y27632), and so on, have demonstrated that ROCK activity plays critical roles in early developmental stages including oocyte maturation, blastocyst formation and implantation (Alarcon and Marikawa 2018; Kawagishi et al. 2004; Kono et al. 2014; Laeno et al. 2013; Marikawa and Alarcon 2019; Saadeldin et al. 2020). A recent review has provided detailed description on the localization of ROCKs and their functions in oocytes and preimplantation embryos in different species (Saadeldin et al. 2020). ROCK activity is also critical for embryonic stem cell (ESC) aggregation and differentiation (see below for more details), which are required for normal tissue morphogenesis including gastrulation and neurulation (Narumiya and Thumkeo 2018; Nishimura et al. 2012; Nishimura and Takeichi 2008; Wei et al. 2001), and cardiac morphogenesis including the movement of second heart field cells, cardiomyocyte proliferation, endocardial cell differentiation and migration, and development of cardiac conduction system (Ellawindy et al. 2015; Hildreth et al. 2009; Vicente-Steijn et al. 2017; Wei et al. 2001, 2002, 2004; Zhao and Rivkees 2003, 2004). Moreover, ROCKs critical roles in development have been reported in vascular remodeling in the yolk sac (Kamijo et al. 2011), lung morphogenesis (McMurtry et al. 2003), brain morphogenesis and development of motor neurons (Kobayashi et al. 2004, 2011; Lin et al. 2013; Zhou et al. 2009), placental development (Thumkeo et al. 2003, 2005), eyelid closure and body wall closure (Duess et al. 2016, 2020; Shimizu et al. 2005; Thumkeo et al. 2005).

Together, the significant involvement of ROCKs in vertebrate developmental processes is to promote actin cytoskeletal organization, actin fiber formation, actomyosin contraction and actin dynamics through intra- and inter-cellular spatial–temporal regulation of ROCKs/MYPT/MLC and ROCKs/LIMK/Cofilin pathways. These pathways are critical for cytokinesis, asymmetric cell division, formation of adherens junctions, apical-basal polarity of epithelial cells, tight junction permeability, cell proliferation, compaction, migration, differentiation and survival (Narumiya and Thumkeo 2018; Saadeldin et al. 2020), showing how ROCK exerts its actions to fit different biological functions. Similar roles of ROCK in tissue morphogenesis, particularly in planar cell polarity, were also observed in drosophila, caenorhabditis elegans, xenopus and zebrafish, all having a single ROCK ortholog, namely ROCK2 (Iida et al. 2020; Kim and Han 2005; Sidor et al. 2020; Tsankova et al. 2017; Wu and Herman 2006). With the advance in our understanding on ROCK signaling during developmental processes, we selected several specific areas using knockout, knockin, and conditional knockout in mice as approaches to provide a detailed review of their current status.

### Genetic Background Can Affect the Developmental Phenotypes and Survival Rates in Systemic ROCK1 and ROCK2 Knockout Mice

Data from our laboratory and others revealed that the genetic background affects the developmental phenotypes and survival rate of *ROCK1*^*–/–*^ embryos (Rikitake et al. 2005; Shimizu et al. 2005; Zhang et al. 2006) (Table 1). *ROCK1*^*–/–*^ mice in C57BL/6 genetic background were born at expected Mendelian ratios, but exhibited eyelids open at birth (EOB) and an omphalocele phenotype due to disorganization of actin filaments in the epithelial cells of the eyelids and in the umbilical ring (Shimizu et al. 2005). The majority of *ROCK1*^*–/–*^ mice (> 90%) die soon after birth due to organs protruding through an omphalocele, such as the liver and gut through the peritoneal cavity. In contrast, the mice in FVB background exhibit a different embryonic phenotype: EOB and omphalocele were absent, nevertheless, the ratio of *ROCK1*^*–/–*^ mice was sub-Mendelian since 60% died in utero before E9.5 (Shi et al. 2011; Zhang et al. 2006). The 40% survival rate was maintained in *ROCK1*^*–/–*^ mice from E9.5 to adult stages suggesting that ROCK1 acts on an early stage of embryonic development prior to organogenesis in FVB background. The reason that genetic background (C57BL/6 vs. FVB) influences the perinatal and early embryonic phenotypes is unknown. In fact, *ROCK1*^+*/–*^ and *ROCK2*^+*/–*^ embryos in FVB background also presented partial premature lethality with 29.1% and 33.8% penetrance, respectively (Table 1). The developmental stages where *ROCK1*^+*/–*^ and *ROCK2*^+*/–*^ embryos died remain to be identified.

**Table 1:** Summary of developmental phenotype and survival rate of ROCK knockout or knockin mice

Genetic background	Genotype	Developmental phenotype and survival rate at weaning age	References
C57BL/6	*ROCK1*^*–/–*^	> 90% perinatal lethal with EOB and ompalocele 3.5–10% survival to adulthood	Rikita et al. (2005), Shi et al. (2011), Shimizu et al. (2005)
	*ROCK1*^*KD/KD*^	~ 100% perinatal lethal with EOB and ompalocele	Shi and Wei (unpublished results)^a^
	*ROCK2*^*–/–*^	~ 80% embryonic lethal from E13.5–E18.5 with placental defects Survival embryos > 90% perinatal lethal with EOB and ompalocele < 1% survival to adulthood	Thumkeo et al. (2003, 2005)
	*ROCK2*^*KD/KD*^	~ 100% embryonic lethal	Shi and Wei (unpublished results)^b^
FVB	*ROCK1*^*–/–*^	~ 60% embryonic lethal before E9.5, ~ 40% survival to adulthood	Zhang et al. (2006)
	*ROCK1*^*KD/KD*^	~ 100% embryonic lethal	Shi and Wei (unpublished results)^c^
	*ROCK1*^+*/–*^	~ 29.1% embryonic lethal, ~ 70.9% survival to adulthood	Shi and Wei (unpublished results)^d^
	*ROCK1*^+*/KD*^	~ 54.5% embryonic lethal ~ 45.5% survival to adulthood	Shi and Wei (unpublished results)^e^
	*ROCK2*^+*/–*^	~ 33.8% embryonic lethal, ~ 66.2% survival to adulthood	Shi and Wei (unpublished results)^f^
	*ROCK2*^+*/KD*^	~ 65% embryonic lethal, ~ 35% survival to adulthood	Shi and Wei (unpublished results)^g^

^a^We analyzed 54 offspring at the age of 3 weeks obtained by intercrossing *ROCK1*^+/KD^ mice (17 *ROCK1*^+/+^, 37 *ROCK1*^+/KD^, 0 *ROCK1*^KD/KD^)

^b^We analyzed 51 offspring at the age of 3 weeks obtained by intercrossing *ROCK2*^+/KD^ mice (16 *ROCK2*^+/+^, 35 *ROCK2*^+/KD^, 0 *ROCK2*^KD/KD^)

^c^We analyzed 61 offspring at the age of 3 weeks obtained by intercrossing *ROCK1*^+/KD^ mice (32 *ROCK1*^+/+^, 29 *ROCK1*^+/KD^, 0 *ROCK1*^KD/KD^)

^d^We analyzed 258 offspring at the age of 3 weeks obtained by crossing *ROCK1*^+/–^ mice with *ROCK1*^+/+^ (151 *ROCK1*^+/+^, 107 *ROCK1*^+/–^)

^e^We analyzed 64 offspring at the age of 3 weeks obtained by crossing *ROCK1*^+/KD^ mice with *ROCK1*^+/+^ (44 *ROCK1*^+/+^, 20 *ROCK1*^+/KD^)

^f^We analyzed 118 offspring at the age of 3 weeks obtained by crossing *ROCK2*^+/–^ mice with *ROCK2*^+/+^ (71 *ROCK2*^+/+^, 47 *ROCK2*^+/–^)

^g^We analyzed 54 offspring at the age of 3 weeks obtained by crossing *ROCK2*^+/KD^ mice with *ROCK2*^+/+^ (40 *ROCK2*^+/+^, 14 *ROCK2*^+/KD^)

The developmental phenotypes of *ROCK2*^*–/–*^ mice also depend on the genetic background (Table 1). *ROCK2*^*–/–*^ mice in a mixed genetic background between 129/SvJ and C57BL/6 demonstrate embryonic lethality due to placental dysfunction from thrombus formation in the labyrinth layer of the placenta, and have intrauterine growth retardation and the majority of embryos (~ 80%) die from E13.5–E18.5 (Thumkeo et al. 2003, 2005). When these *ROCK2*^*–/–*^ mice were backcrossed into a C57BL/6 genetic background, they exhibit not only the placental phenotype but also perinatal EOB and omphalocele phenotype (Thumkeo et al. 2005), indicating that genetic background affects the EOB and omphalocele phenotype in *ROCK2*^*–/–*^ mice.

Due to the high degree of penetrance, the viability of *ROCK1*^*–/–*^ and *ROCK2*^*–/–*^ mice in C57BL/6 background is extremely low. The shared perinatal EOB and omphalocele phenotypes in these mice suggest that they act functionally redundant in these closure processes through ROCKs/MYPT/MLC pathway regulating the assembly of actin bundles essential for closure of eyelid and ventricular body wall in mouse embryos (Shimizu et al. 2005). Remarkably, deletion of LIMK2, a key downstream effector of ROCKs, also caused the EOB phenotype due to inhibition of keratinocyte migration during eyelid formation (Rice et al. 2012), supporting the contribution of ROCKs/LIMK2/Cofilin pathway to this developmental process. On the other hand, the shared partial embryonic lethality of *ROCK1*^*–/–*^, *ROCK1*^+*/–*^ and *ROCK2*^+*/–*^ in FVB background also points that ROCK1 and ROCK2 have shared functions. A shared characteristic in *ROCK1*^*–/–*^ and *ROCK2*^*–/–*^ mice, regardless of their genetic background, is that they seem to develop normally, healthy and fertile after surviving their intrauterine and perinatal period (Rikitake et al. 2005; Shimizu et al. 2005; Thumkeo et al. 2003; Zhang et al. 2006), suggesting that each isoform is able to functionally compensate in vivo for the loss of the other during the remaining developmental processes. In addition, there is no compensatory up-regulation of the ROCK1 expression in *ROCK2*^*–/–*^ mice and vice versa. Together, these genetic studies using *ROCK1*^*–/–*^ and *ROCK2*^*–/–*^ mice provide significant insights into the biological functions of ROCK1 and ROCK2 isoforms which appear to be largely redundant during development.

### Can ROCK1 and ROCK2 Kinase-Dead Knockin Mice Phenocopy Knockout Mice?

**Fig. 2:**
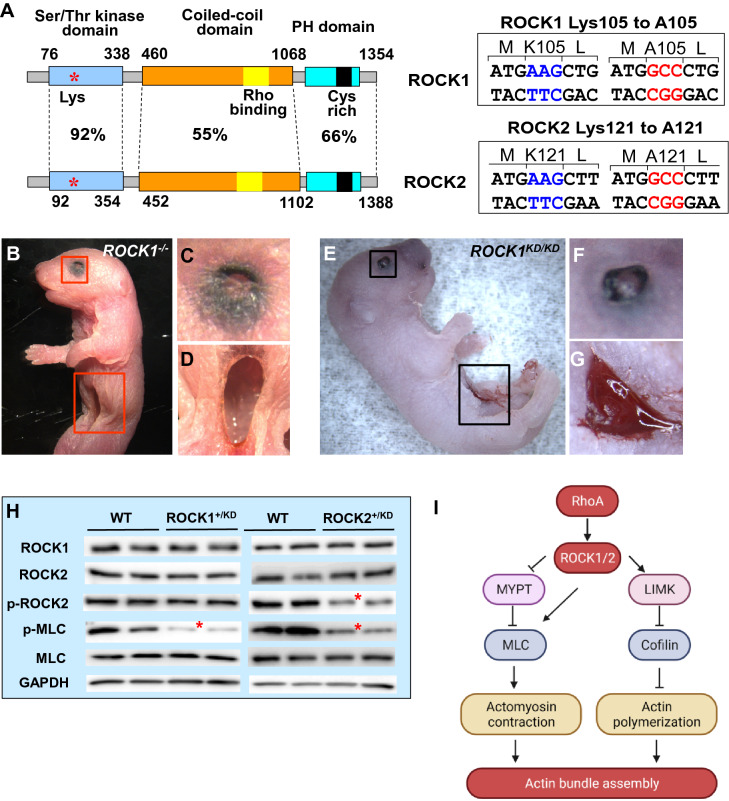
Characterization of ROCK1 or ROCK2 KD knockin mice. **A** Molecular structure of ROCK1 and ROCK2. Lysine (Lys or K) 105 in ROCK1 catalytic kinase domain or Lysine121 in ROCK2, required for ATP binding, is exchanged for Alanine (Ala or A) in ROCK1 or ROCK2 KD knockin mice. *PH* pleckstrin-homology. **B**–**G** *ROCK1*^KD/KD^ mice reproduce the perinatal phenotype of *ROCK1*^*–/–*^ mice in C57BL/6 background. **B** Left side view of a dead *ROCK1*^*–/–*^ neonate, with left eye open (**C**) and umbilical region open (**D**). **E** Right side view of a live *ROCK1*^KD/KD^ neonate, with right eye open (**F**) and umbilical region open (**G**). **H** Representative image of Western blot performed with *ROCK1*^+/KD^ and *ROCK2*^+/KD^ MEFs. One ROCK1-KD allele achieves reduction of p-MLC level, but has no inhibitory effect on ROCK2 activity monitored by p-ROCK2(Ser1366). One ROCK2-KD allele achieves reduction of p-ROCK2 and p-MLC. *p < 0.05 vs. WT cells under same condition. **I** Simplified scheme depicting the actions of ROCKs in eye lid and umbilical ring epithelial cells

ROCK isoform deletion removes both kinase-dependent and independent functions of the ROCK protein. To compare the effects of genetic deletion to those of genetic inactivation, we have analyzed the developmental phenotypes of ROCK isoform kinase-dead (KD) point-mutation knockin mice (Shi and Wei, unpublished results) (Fig. 2); mice were generated in the C57BL/6 background by Merck Research Laboratories (available through Taconic; Rock1—Model 12904—PM; Rock2—Model 12979—PM). Since the inactivated ROCK isoforms are still able to interact with their regulators such as RhoA and with downstream substrates, they can competitively inhibit other RhoA effectors behaving as dominant negatives. Viewed in this way, KD knockin mutation may not be functionally identical to null mutation. Molecular analyses indicate that the KD mutation in each isoform inactivates kinase activity, but has no detectable changes in protein expression (Wei et al. 2020) (Fig. 2H). However, when the heterozygous *ROCK1*^+/KD^ mice were intercrossed, no viable *ROCK1*^KD/KD^ mouse at birth was obtained. Similarly, no viable *ROCK2*^KD/KD^ mouse at birth was obtained when the heterozygous *ROCK2*^+/KD^ mice were intercrossed (Table 1). The high premature death rate of *ROCK1*^KD/KD^ and *ROCK2*^KD/KD^ mice (~ 100%) in C57BL/6 background is similar to that of *ROCK1*^*–/–*^ and *ROCK2*^*–/–*^ mice (95–100%), suggest that ROCK isoform kinase inactivation in mice may recapitulate major developmental phenotypes of ROCK isoform deficient mice. To answer whether *ROCK1*^KD/KD^ mice reproduce the developmental phenotypes reported in *ROCK1*^*–/–*^ mice such as EOB and omphalocele, we analyzed the genotype distribution at birth. Findings showed that the number of the *ROCK1*^KD/KD^ neonates (12 out of 51; 23.5%) is close to the expected Mendelian ratio (25%), but they all presented with EOB and omphalocele perinatal phenotype (Fig. 2B) and died within few hours after birth. These results support the notion that ROCK1-KD allele can phenocopy the perinatal phenotype of ROCK1-null allele in C57BL/6 background.

The high premature mortality rates in both knockout and KD mice of C57BL/6 background make it difficult to evaluate if KD alleles have dominant negative effects compared to the knockout allele. However, the partial lethality of *ROCK1*^*–/–*^ (60%), *ROCK1*^+/–^ (29.1%), and *ROCK2*^+/–^ (33.8%) mice in FVB background have provided a well-defined system for further evaluating the impact of KD mutation in ROCK1 or ROCK2 endogenous gene (Table 1). Following both ROCK1-KD and ROCK2-KD alleles were backcrossed into the FVB background (> 10 generations), *ROCK1*^KD/KD^ mice show significantly increased mortality compared to *ROCK1*^*–/–*^ mice (100% vs. 60%), suggesting that two copies of ROCK1-KD allele in *ROCK1*^KD/KD^ mice produce dominant negative effects over the two preserved ROCK2 alleles during early embryogenesis. In addition, *ROCK1*^+/KD^ and *ROCK2*^+/KD^ mice showed increased embryonic mortality rates compared to *ROCK1*^+/–^ (54.5% vs. 29.1%) and *ROCK2*^+/–^ mice (65% vs*.* 33.8%), suggesting that one copy of ROCK1-KD or ROCK2-KD allele in *ROCK1*^+/KD^ or *ROCK2*^+/KD^ mice produce dominant negative effects over the preserved ROCK1 and ROCK2 alleles, further supporting the notion that the KD allele acts as a dominant negative during early embryogenesis in FVB background.

To summarize the genetic validation of the KD alleles in comparison with the null alleles, two main points reveal potential context-dependent KD allele functions: (1) KD-allele is functional identical to the null-allele that is supported by the fact that *ROCK1*^KD/KD^ mice reproduce perinatal lethal phenotypes of *ROCK1*^*–/–*^ mice in C57BL/6 background through suppressing ROCKs/MYPT/MLC and ROCKs/LIMK/Cofilin pathways in eye lid and umbilical ring epithelial cells (Fig. 2I); (2) KD-allele produces dominant negative effects to wild-type alleles, which is supported by the fact that, in comparison among *ROCK1*^*–/–*^, *ROCK1*^+/–^ and *ROCK2*^+/–^ mice of FVB background, *ROCK1*^KD/KD^, *ROCK1*^+/KD^ and *ROCK2*^+/KD^ mice all showed increased embryonic mortality rates (Table 1).

### What Can We Learn from the Deletion of Both ROCK1 and ROCK2 Isoforms in Various Mouse Developmental Stages?

To determine the impact of the complete removal of ROCK activity on the mouse development, we generated double ROCK deletion mice via a series of cell type-specific Cre mice (Shi and Wei, unpublished results) (Table 2). Our first endeavor was to generate α-myosin heavy chain (MHC)-Cre/*ROCK1*^f/f^/*ROCK2*^f/f^ mice characterized by cardiomyocyte-specific ROCK knockout via MHC-Cre (Agah et al. 1997) which shows a developmental stage-specific expression of endogenous MHC: before birth it is expressed in the developing heart from E8.0 and largely restricted to the atrium; 2 days after birth, the expression is markedly up-regulated in the atrium and ventricle (Ng et al. 1991; Subramaniam et al. 1991). Both MHC-Cre/*ROCK1*^f/f^/*ROCK2*^+/f^ and MHC-Cre/*ROCK1*^+/f^/*ROCK2*^f/f^ mice retaining one wild-type allele, either ROCK1 or ROCK2 gene, were viable up to 12 months of age, but no viable MHC-Cre/*ROCK1*^f/f^/*ROCK2*^f/f^ mouse was obtained in postnatal pups through crossing MHC-Cre/*ROCK1*^f/f^/*ROCK2*^+/f^ or MHC-Cre/*ROCK1*^+/f^/*ROCK2*^f/f^ mice with *ROCK1*^f/f^/*ROCK2*^f/f^ mice. These results indicate that complete removal of both ROCK1 and ROCK2 in cardiomyocytes leads to embryonic lethality, at least one copy of ROCK isoform genes is required in this cell type during cardiac development. Surprisingly, mice with MHC-Cre-mediated cardiomyocyte-specific deletion of RhoA, the shared activator of ROCKs, can survive to adulthood without basal pathological phenotype (Lauriol et al. 2014). Two probable explanations can help to understand the absence of requirement for RhoA in cardiomyocytes during development: (1) ROCKs can also be activated by other Rho family members, for instance RhoB and RhoC (Fujisawa et al. 1996), therefor ROCK activity is not completely abolished in the RhoA knockout cardiomyocytes; (2) since only one out of four copies of ROCK isoform genes is required for normal cardiac morphogenesis, RhoA deletion is not sufficient to reduce ROCK activity below the threshold.

**Table 2:** Viability of cell type-specific ROCK1 and/or ROCK2 knockout mice at weaning ages in C57BL/6 background

Cell type-specific Cre	*ROCK1*^f/f^	*ROCK2*^f/f^	*ROCK1*^+/f^ *ROCK2*^+/f^	*ROCK1*^+/f^ *ROCK2*^f/f^	*ROCK1*^f/f^ *ROCK2*^+/f^	*ROCK1*^f/f^ *ROCK2*^f/f^
Cardiomyocyte (MHC)	Viable	Viable	Viable	Viable	Viable	No^a^
Fibroblasts (Peri)	Viable	Viable	Viable	Viable	Viable	No^b^
Endothelial (Tie2)	Viable	Viable	Viable	No^c^	No^c^	No^c^
Endocardial and cardiomyocyte (Nkx2.5)	Viable	Viable	Viable	No^d^	No^d^	No^d^
Neural crest (Wnt1)	Viable	Viable	Viable	No^e^	No^e^	No^e^

^a^We crossed MHC-Cre/*ROCK1*^f/f^/*ROCK2*^+/f^ or MHC-Cre/*ROCK1*^+/f^/*ROCK2*^f/f^ mice with *ROCK1*^f/f^/*ROCK2*^f/f^ mice, no viable MHC-Cre/*ROCK1*^f/f^/*ROCK2*^f/f^ mouse was obtained after screening over 200 mice after birth (Shi and Wei, unpublished results)

^b^We crossed Peri-Cre/*ROCK1*^f/f^/*ROCK2*^+/f^ or Peri-Cre/*ROCK1*^+/f^/*ROCK2*^f/f^ mice with *ROCK1*^f/f^/*ROCK2*^f/f^ mice, no viable Peri-Cre/*ROCK1*^f/f^/*ROCK2*^f/f^ mouse was obtained after screening over 100 mice after birth (Shi and Wei, unpublished results)

^c^We crossed Tie2-Cre/*ROCK1*^+/f^/*ROCK2*^+/f^ with *ROCK1*^f/f^/*ROCK2*^f/f^ mice, no viable Tie2-Cre/*ROCK1*^f/f^/*ROCK2*^+/f^, Tie2-Cre/*ROCK1*^+/f^/*ROCK2*^f/f^ or Tie2-Cre/*ROCK1*^f/f^/*ROCK2*^f/f^ mouse was obtained after screening over 100 mice after birth (Shi and Wei, unpublished results)

^d^We crossed Nkx2.5-Cre/*ROCK1*^+//f^/*ROCK2*^+/f^ mice with *ROCK1*^f/f^/*ROCK2*^f/f^ mice, no viable Nkx2.5-Cre/*ROCK1*^f/f^/*ROCK2*^+/f^, Nkx2.5-Cre/*ROCK1*^+/f^/*ROCK2*^f/f^ or Nxk2.5-Cre/*ROCK*1^f/f^/*ROCK2*^f/f^ mouse was obtained after screening over 100 mice after birth (Shi and Wei, unpublished results)

^e^We crossed Wnt1-Cre/*ROCK1*^+/f^/*ROCK2*^+/f^ mice with *ROCK1*^f/f^/*ROCK2*^f/f^ mice, no viable Wnt1-Cre/*ROCK1*^f/f^/*ROCK2*^+/f^, Wnt1-Cre/*ROCK1*^+/f^/*ROCK2*^f/f^ or Wnt1-Cre/*ROCK1*^f/f^/*ROCK2*^f/f^ mouse was obtained after screening over 100 mice after birth (Shi and Wei, unpublished results)

In addition, we generated Periostin (Peri)-Cre/*ROCK1*^f/f^/*ROCK2*^f/f^ mice to make constitutive fibroblast-specific knockout via Peri-Cre (Table 2). Periostin, a matricellular protein, is initially detected in cardiac fibroblasts at E10.0 as well as the nascent endocardial cushions, and is robustly induced in activated fibroblasts (myofibroblasts) in response to stress in adult hearts (Kaur et al. 2016; Oka et al. 2007; Snider et al. 2009). Both Peri-Cre/*ROCK1*^f/f^*/ROCK2*^+/f^ and Peri-Cre/*ROCK1*^+/f^/*ROCK2*^f/f^ mice retaining one wild-type allele, either ROCK1 or ROCK2 gene, were viable up to 12 months of age, but no viable Peri-Cre/*ROCK1*^f/f^/*ROCK2*^f/f^ mouse was obtained in postnatal pups through crossing Peri-Cre/*ROCK1*^f/f^/*ROCK2*^+/f^ or Peri-Cre/*ROCK1*^+/f^/*ROCK2*^f/f^ mice with *ROCK1*^f/f^/*ROCK2*^f/f^ mice. The results indicate that removal of both ROCK1 and ROCK2 in periostin expressing cells during development will result in embryonic lethality as we observed in MHC expressing cells, therefore indicating one copy of ROCK isoform genes is required in this cell type during development.

Beyond MHC-Cre and Peri-Cre, we endeavored to generate Tie2-Cre/*ROCK1*^f/f^/*ROCK2*^f/f^, Nkx2.5-Cre/*ROCK1*^f//f^/*ROCK2*^f/f^, and Wnt1-Cre/*ROCK1*^f//f^/*ROCK2*^f/f^ mice through the same breeding strategy as described above (Table 2). In Tie2-Cre mice, Tie2 promoter drives Cre expression specifically in endothelial cells from E7.5 throughout development to adulthood (Kisanuki et al. 2001). Nkx2.5-Cre provides Cre-mediated recombination initially in the cardiac progenitor cells that form the cardiac crescent at E7.5, and continues throughout development in cardiomyocytes (Moses et al. 2001). Wnt1-Cre is active from E8.0 in the neural plate prior to the emigration of the neural crest, which contributes to a variety of developmental processes including craniofacial structure and cardiac outflow tract formation (Jiang et al. 2000). In brief, no viable double ROCK deficient mouse was obtained from these crossings, indicating that complete removal of both ROCK1 and ROCK2 in these Tie2, Nkx2.5, and Wnt1 expressing cells will lead to embryonic lethality. It is worthy to note, compared to MHC-Cre- and Peri-Cre-mediated removal of ROCK1 and ROCK2, in Tie2-Cre, Nkx2.5-Cre, and Wnt1-Cre involved breeders, no viable homo-heterozygous *ROCK1*^f/f^*/ROCK2*^+/f^ or *ROCK1*^+/f^*/ROCK2*^f/f^ mouse was obtained in postnatal pups, indicating that two copies of ROCK isoform genes are required in the Tie2, Nkx2.5, and Wnt1 expressing cells during development. The early embryonic lethality caused by homo-heterozygous ROCK deficiency in endothelial cells in Tie2-Cre/*ROCK1*^f/f^*/ROCK2*^+/f^ and Tie2-Cre*ROCK1*^+/f^*/ROCK2*^f/f^ mice is consistent with the previous observations showing that the homo-heterozygous *ROCK1*^*–/–*^*/ROCK2*^+/–^ or *ROCK1*^+/-^/*ROCK2*^-/-^ mice die in utero during E9.5–12.5 due to defective vascular remodeling in the yolk sac, and that both ROCK isoforms are expressed in endothelial cells in the yolk sac at E9.5 (Kamijo et al. 2011). In addition, the early embryonic lethality caused by homo-heterozygous ROCK deficiency in neural crest cells in Wnt1-Cre/*ROCK1*^f/f^*/ROCK2*^+/f^ and Wnt1-Cre*ROCK1*^+/f^*/ROCK2*^f/f^ mice is consistent with an early study reporting that Wnt1-Cre mediated expression of a ROCK dominant negative mutant caused severe craniofacial malformation and severe cardiac outflow malformation in mouse embryos (Phillips et al. 2012).

The findings from above described studies on five constitutive deletion of both ROCK1 and ROCK2 by cell type-specific expressed Cre have shown that either removal of four copies of ROCK isoform genes in all these cell types or three out of four copies in some of these cell types will lead to embryonic lethality. These observations support an essential role of ROCK activity after the onset of Cre expression from E7.5 (Tie2, Nkx2.5), E8.0 (Wnt1, MHC), and E10.0 (Peri) in these specific cell types during development. The early embryonic lethality of cell type-specific double ROCK knockout mice, together with the embryonic and perinatal phenotypes of systemic single or double ROCK1 and ROCK2 knockout mice, support the notion that the copy number requirement of ROCK isoform genes is dependent on the cell type, genetic background and developmental stage (Table 3). Moreover, homo-homozygous *ROCK1*^*–/–*^/*ROCK2*^*–/–*^ mice die in utero from E3.5 to E9.5 (Kamijo et al. 2011) supporting the notion that global ROCK activity critically contributes to embryogenesis from E3.5, earlier than the cell type-specific ROCK activity. To bypass the critical developmental stages while achieving global or cell type-specific double ROCK deletion, the inducible approach via inducible Cre recombinase, e.g. through tamoxifen or interferon, is an appropriate approach for double ROCK knockout. We have achieved double ROCK isoform deletion in cardiomyocytes (Shi et al. 2019) using tamoxifen-inducible MHC-Cre (Sohal et al. 2001), in stress-activated fibroblasts using tamoxifen-inducible Peri-Cre (Kanisicak et al. 2016; Khalil et al. 2017) and in blood cells using interferon-inducible Mx-Cre (Kuhn et al. 1995). These mice are viable after inducible deletion of double ROCK isoforms (Shi et al., unpublished results).

**Table 3:** Summary of copy number requirement of ROCK isoform genes associated with cell types and developmental stages

Required copy	Cell type	Genotype	Lethal stage	References
0	Cardiomyocytes	Inducible MHC-Cre/*ROCK1*^f/f^/*ROCK2*^f/f^	Not lethal	Shi et al. (2019)
1	Cardiomyocytes	MHC-Cre/*ROCK1*^f/f^/*ROCK2*^f/f^	After E8.0	Shi and Wei (unpublished results)^a^
	Fibroblasts	Peri-Cre/*ROCK1*^f/f^/*ROCK2*^f/f^	After E10.0	Shi and Wei (unpublished results)^a^
	Unknown	*ROCK1*^*–/–*^/*ROCK2*^*–/–*^	E3.5 – 9.5	Kamijo et al. (2011)
2	Endothelial cells	Tie2-Cre/*ROCK1*^+/f^/*ROCK2*^f/f^; Tie2-Cre/*ROCK1*^f/f^/*ROCK2*^+/f^	After E7.5	Shi and Wei (unpublished results)^a^
		*ROCK1*^+/–^/*ROCK2*^*–/–*^; *ROCK1*^*–/–*^/*ROCK2*^+/–^	E9.5 – 12.5	Kamijo et al. (2011)
	Cardiac progenitors	Nkx2.5-Cre/*ROCK1*^+/f^/*ROCK2*^f/f^; Nkx2.5-Cre/*ROCK1*^f/f^/*ROCK2*^+/f^	After E7.5	Shi and Wei (unpublished results)^a^
	Neural plate	Wnt1-Cre/*ROCK1*^+/f^/*ROCK2*^f/f^; Wnt1-Cre/*ROCK1*^f/f^/*ROCK2*^+/f^	After E8.0	Shi and Wei (unpublished results)^a^
3	Eye lid and umbilical ring epithelial cells	*ROCK1*^*–/–*^ (C57); *ROCK1*^+/–^/*ROCK2*^+/–^ (C57); *ROCK2*^*–/–*^ (C57)	Perinatal	Rikita et al. (2005), Shimizu et al. (2005), Shi et al. (2011), Thumkeo et al. (2005)
4	Unknown	*ROCK1*^+/–^ (FVB); *ROCK2*^+/–^ (FVB)	Before E9.5	Zhang et al. (2006); Shi and Wei (unpublished results)^b^

^a^See Table 2

^b^See Table 1

In addition to bypassing the embryonic development stages where at least one or two copies of ROCK isoform genes in specific cell types is required, the approach through inducible Cre-mediated deletion of double ROCK isoforms is useful for precisely delineating the roles of ROCKs in postnatal tissue development and in the initiation and progression of diseases. Regarding postnatal heart development, data from our laboratory and others have revealed that there is a functionally intact caspase-dependent death machinery in neonatal hearts that is rapidly silenced within the first 3 weeks of postnatal time window (Madden et al. 2007; Shi et al. 2012), indicating that neonatal hearts are more susceptible to cardiotoxicity induced by chemotherapeutic agents or to genetically induced cardiomyopathy. ROCK1 deficiency exhibited cardio-protection in various injury models (Chang et al. 2006; Shi et al. 2008, 2010, 2011, 2018; Yue et al. 2014; Zhang et al. 2006) including those neonatal hearts affected by genetic cardiomyopathy due to the robust upregulation of MHC promoter-mediated transgene expression (Shi et al. 2008, 2010; Yang et al. 2012). It will be of interest to assess the impact of double ROCK isoform deletion on postnatal heart maturation window and on cardiac apoptosis induced by chemotherapeutic agents in immature hearts.

It is fascinating to understand the different functions of ROCKs in early developmental, postnatal developmental and mature heart stages, especially in disease conditions of adult life. Three major differences are interesting: (1) both ROCK isoforms act functionally redundant in many developmental processes, for instance in the closure processes of eye lid and umbilical ring (Fig. 2), but they exhibit distinct roles in many circumstances of adult pathophysiology; (2) the gene copy number requirement can be developmental stage- and cell type-dependent, for instance at least one copy is required in cardiomyocytes of developing heart for normal cardiac morphogenesis, but their complete deletion is cardioprotective at least during aging (Table 3); (3) ROCK isoform expression can be differently regulated, for instance there is no compensatory up-regulation of the ROCK2 expression in *ROCK1*^*–/–*^ mice during embryonic development, but up-regulation of ROCK2 was observed in ROCK1-deficient cardiomyocytes in adult heart (Shi et al. 2019).

## ROCKs Play Critical Roles in Stem Cell Renewal and Differentiation

In addition to the critical roles of ROCKs in embryonic morphogenesis, a rapid growing research area related to ROCKs in development is stem cell development covering embryonic stem cell patterning, cell lineage commitment of mesenchymal stem cells (MSCs), therapeutic applications of stem cells derived from adult tissues with self-renewal and multi or pluripotent abilities, and the extensively applications of ROCK inhibitors in stem cell culture systems, for instance conditional reprogramming.

### Conditional Reprogramming

There has been a strong push to expand primary culture of mammalian cells including stem cells for a broad spectrum of applications, specifically in disease modeling, drug discovery and evaluation, regenerative medicine and precision medicine. Conditional reprogramming technology involves co-culture of irradiated mouse fibroblast feeder cells and digested primary normal or pathogenic epithelial cells in the presence of the ROCK inhibitors (Y-27632). This changes the external culture environment to allow cells to acquire stem-like characteristics, e.g., capable of long-term expansion in vitro, while retain their ability to fully differentiate (Chapman et al. 2014; Liu et al. 2012, 2017; Wu et al. 2020). Both ROCK inhibitors and feeder cells are essential for long-term expansion of primary cells. However, only feeder cells or ROCK inhibitors cannot support a long-term expansion of primary cells that become senescent after a few passages (Wu et al. 2020). Compared to other techniques aimed at expanding stem-like cells and maintaining their pluripotency, conditional reprogramming is easy to operate in the laboratory, maintain cell genome stability while keeping differentiation potential, and avoid genomic manipulation as well as caused ethical issue. Thus, benefit from conditional reprogramming, primary epithelial cells derived from almost all primary tissue samples (e.g., adult and embryonic) can be cultured and expanded. ROCK inhibitors have been broadly used for primary cells in vitro achieving conditional reprogramming. The underlying mechanisms responsible for this phenomenon include increased cell cycle progression and suppressed senescence (Chapman et al. 2014; Ligaba et al. 2015), blockade of actomyosin hypercontraction-, Myc- or p53-mediated apoptosis (Dakic et al. 2016; Koyanagi et al. 2008; Kurosawa 2012; Mondal et al. 2018; Ohgushi et al. 2010; Watanabe et al. 2007), suppression of NOTCH-, WNT5A- or TGFβ/SMAD-induced differentiation (Ligaba et al. 2015; Santos et al. 2010; Yugawa et al. 2013), maintenance of stem-like properties through up-regulation of related stem cell makers (Suprynowicz et al. 2012, 2017), and promotion of cell-extracellular matrix and cell–cell communication (Reynolds et al. 2016).

### Inhibition of ROCK Activity Can Augment Stem Cell Renewal

Prior to the application of conditional reprogramming technology, ROCK inhibition was initially found able to facilitate the in vitro growth of pluripotent human ESCs (hESCs) due to inhibiting dissociation-induced apoptosis via blockage of ROCK/MLC regulated actomyosin contraction (Koyanagi et al. 2008; Kurosawa 2012; Ohgushi et al. 2010; Watanabe et al. 2007). ROCK inhibitors not only protect hESCs from apoptosis during culturing but also increase recovery and colony formation after freeze-thawing from a cryopreserved sample (Baharvand et al. 2010; Claassen et al. 2009; So et al. 2020). ROCK inhibitors can keep hESCs and human-induced pluripotent stem cells (hiPSCs) undifferentiated in culture and induce metabolic changes in these cells (Vernardis et al. 2017). Therefore, supplementing cell culture with ROCK inhibitors has been proven to be a simple, efficient, and versatile approach for the development of new protocols of hiPSC culture on a large scale (Rivera et al. 2020), transportation/shipment of various types of stem cells (Ye et al. 2020), improvement of derivation methods for mouse ESCs (mESCs) (Zhang et al. 2012) and for handling porcine ESCs and PSCs (Baek et al. 2019).

### Stem Cell Differentiation and Therapeutic Applications

Human PSCs, including hESCs and hiPSCs, have the ability for self-renewal and differentiation into any somatic cell making them significant in both translational research of regenerative medicine and potential therapeutic applications. ROCK inhibitors were proven to not only protect hESCs through inhibiting dissociation-induced apoptosis during culture as mentioned above, but also increase survival of hESC-derived cardiomyocytes after dissociation, therefore allowing production of specialized cell types for the generation of disease models and for cell replacement therapy (Braam et al. 2010). Among all types of adult stem cells, multipotent MSCs are of great interest to cell-based therapies because of their easy isolation and high proliferative rate in vitro. Inhibition of ROCK in human bone marrow-derived MSCs facilitates their differentiation into keratinocyte-like cells, and promotes the proliferation and survival of human primary keratinocytes, which can be beneficial for patients with burns, trauma, or disease (Li et al. 2015). Furthermore, ROCK inhibitors show benefits in maintaining multilayered proliferation of confluent human bone marrow-derived MSCs and their potency to differentiate into osteoblast and adipocyte lineages, which could be useful in periodontal tissue regeneration (Nakamura et al. 2014). ROCK inhibition reduces stress induced by mechanotransduction of human bone marrow-derived MSCs, the latter can increase secretion and blood concentration of stanniocalcin-1, and support cell survival and angiogenesis (Zonderland et al. 2020). Manipulation of human umbilical cord MSCs with ROCK inhibitors improved viability and transfection efficiency, and enhanced the utility of differentiation and reprogramming of the cells, which is beneficial for tissue engineering applications (Mellott et al. 2014). Urine-derived stem cells (UDSC) in humans are considered as desirable sources for cell therapy because donor-specific UDSC are easily and non-invasively obtained and these cells can be reprogramed into hiPSCs. ROCK inhibitors, together with Matrigel and flavonoids could improve UDSC isolation, proliferation, and differentiation potency (Kim et al. 2020). Stem cells from human exfoliated deciduous teeth (SHED) are multipotent stem cells with neural crest cell origin. The addition of ROCK inhibitors in the culture medium could enhance the viability of SHED, and if treating the cells with the combination of ROCK inhibitors and Noggin, it would further synergistically promote their differentiation into neuron-like cells and provide a promising source of stem cells for neurodegenerative disease treatment (Yang et al. 2020). The development of hiPSCs from fibroblasts of patients could offer a possibility of developing individualized treatment plans. ROCK inhibition up-regulates nuclear receptor NR4A1 and promotes phenotypic rescue in neurons differentiated from hiPSCs derived from fibroblasts of patients with oligophrenin-1 loss of function. Oligophrenin-1 loss of function is responsible for X-linked intellectual disability, and ROCK inhibition research can be used to provide a model for this neural disease and its treatment (Compagnucci et al. 2016).

In animal stem cell studies, inactivation of ROCK promotes multipotent MSC differentiation into epithelial cells for airway repair/remodeling through the WNT (Wingless and Int-1) signaling factor LEF1 in a chronic asthma mouse model, therefore providing a novel therapeutic target for patients with asthma (Ke et al. 2019). A study performed in 2D matrigel culture system supplemented of ROCK inhibitors and VEGF-A has shown that mESC-derived Flk1+ mesodermal precursor cells produced endothelial cells at high purity, providing a potential strategy for therapeutic neovascularization (Joo et al. 2012). Regarding neuronal differentiation, suppression of ROCK promoted the differentiation of mESCs into neurons via activating phosphatidylinositol 3-kinase signaling pathway (Kamishibahara et al. 2016). Furthermore, ROCK inhibition rescued neurogenesis of rat hippocampal neural stem cells cultured on stiff substrates through reducing stiffness-induced myosin contractility and nuclear translocation of angiomotin resulting in increased β-catenin activity (Kang et al. 2020). In contrast to their roles of promoting differentiation described above, ROCK inhibition also has roles in suppressing differentiation in other cell types. For example, the inhibition of ROCK suppressed mechanical tension-induced osteogenic differentiation of rat cranial sagittal suture MSCs, down-regulated TAZ expression and inhibited nuclear translocation that is involved in osteogenic differentiation (Li et al. 2020).

To study embryonic patterning, the addition of ROCK inhibitors in protocol allowed in vitro differentiating rat ESCs into embryoid bodies which further propagated and differentiated into three embryonic germ layers and functional cardiomyocytes (Cao et al. 2011). In cultured hiPSCs, ROCK activity could regulate mesodermal spatial organization and subsequent vascular fate of the cells which were differentiated into endothelial cells (low cytoskeletal tension and high cell–cell contact) vs. pericytes (high cytoskeletal tension and low cell–cell contact) and self-organized to form blood vessels (Smith et al. 2018). Moreover, studies employing CRISPR gene editing induced specific knockdown of ROCK1 in subpopulations of hiPSC colonies within an otherwise homogeneous population of pluripotent cells. The resulting mosaic knockdown of ROCK1 triggered cellular self-organization within colonies due to the cells lacking ROCK1 moving to the periphery of the colonies while retaining an epithelial pluripotent phenotype, which supports that ROCK1 is a significant player in tissue development and cell organization processes (Libby et al. 2018, 2021).

Regarding cell lineage commitment, RhoA/ROCK-mediated cell–cell and cell–matrix interactions have been profoundly involved in osteogenesis, myogenesis, adipogenesis of progenitor cells and stem cells for both physiology and pathophysiology. For instance, in embryonic heart, ROCK inhibition could lead to fibrofatty replacement of cardiomyocytes in the right ventricle of adult mice, but this event could not be detected after birth (Ellawindy et al. 2015). In line with this observation, ROCK inhibition primed embryonic cardiac progenitors to be ready to switch to the brown/beige adipocyte lineage in response to adipogenesis-inducing signals, supporting that RhoA/ROCK-mediated actin cytoskeleton dynamics control an active MRTF/SRF transcriptional program essential for cardiomyocyte identity during cardiomyocyte differentiation (Dorn et al. 2018). In addition to the determination between cardiomyocyte and adipocyte lineages, ROCK activity is also involved in the determination between white and brown/beige adipocyte formation via control of MRTF/SRF transcriptional program in adult fat tissues (McDonald et al. 2015; Nobusue et al. 2014). We recently discovered that ROCK2 inhibition enhances beige adipogenesis of stromal-vascular cells and subcutaneous white adipose tissue in mice; the course is associated with increased thermogenic program in white and brown fat tissue (Wei et al. 2020).

### Cancer Stem Cells and Precision Medicine

Accumulating evidence supports the concept that ROCK plays vital roles in tumor development and progression through regulating many key cellular functions associated with malignancy, including tumorigenicity, tumor growth, metastasis, angiogenesis, tumor cell apoptosis/survival and chemoresistance. Since ROCK has emerged as a promising target for the development of novel anti-cancer drugs, the prospect of applying ROCK inhibition to delay and block tumorigenicity, tumor growth, tumor cell invasion and metastasis has been extensively evaluated (de Sousa et al. 2020; Shahbazi et al. 2020; Wei et al. 2016). Although ROCK activation is generally considered to be oncogenic, some studies show ROCK functions as a negative regulator in cancer progression. The possibilities for the observed contradictory contributions of ROCK signaling to cancer progression include the complexities of cell context and microenvironment, potential compensatory up-regulation of ROCK isoform triggered by the inhibition of another isoform, and compensatory effects of other signaling pathways. A recent study has shown that while the loss of either ROCK1 or ROCK2 had no negative impact on tumorigenesis in mouse models of non-small cell lung cancer and melanoma, the loss of both isoforms blocked tumor formation owing to inhibiting cell cycle progression and tumorigenesis (Kumper et al. 2016). This approach through double ROCK isoform deletion can help future studies to further clarify the precise roles of ROCK isoforms affecting specific types of cancer processes.

Another potential explanation for contradictory contributions of ROCK signaling to cancer progression is relating to the pro-survival and proliferative effects of ROCK inhibition on cancer stem cells, dormant cells, and circulating tumor cells which are responsible for cancer cell dissemination and metastasis after ROCK inhibition (de Sousa et al. 2020; Wei et al. 2016). Cancer stem cells, also named tumor-initiating cells, represent a small subpopulation of cancer cells with self-renewable and multi or pluripotent abilities. ROCK inhibition increased adhesion of cancer stem cells from primary human glioblastoma to soft extracellular matrix leading to increased migration and tissue invasion (Wong et al. 2015). In contrast, ROCK inhibition in stromal cells surrounding cancer stem cells reduced stiffness of extracellular matrix leading to reduced stem cell adhesion to extracellular matrix and consequently reduced spreading, migration, and proliferation (Choi et al. 2015). Furthermore, ROCK inhibition in dormant MCF-7 breast cancer cells disrupted cell junction, promoted cell proliferation, migration and invasion associated with increased Rac GTPase-mediated signaling activation (Yang and Kim 2014). Likewise, exposure of the circulating breast cancer cells to ROCK inhibitors destabilized the actin cortex and increased the formation of microtentacles which are microtubule-based structures and can enhance their reattachment efficacy to the vasculature and accordingly, their metastatic potential (Bhandary et al. 2015). Hence, anticipated effects of ROCK inhibition relevant pro-survival and pro-extracellular matrix adhesive should be measured to avoid potential undesirable effects before ROCK inhibition as a therapeutic strategy in any type of cancer treatment.

Similar to the culture of stem cells of non-cancer origins, the inclusion of ROCK inhibitors has also become part of standard stem cell culture protocols for cancer stem cells (Castro et al. 2013; Lin et al. 2018; Ohata et al. 2012; Tilson et al. 2015). In addition to promoting survival, ROCK inhibitors also increased proliferation of cancer stem cells (Castro et al. 2013), and enhanced stem-like phenotypes with increased expression of related stem cell markers (Ohata et al. 2012; Tilson et al. 2015). Furthermore, conditional reprogramming technology characterized by co-culture with ROCK inhibitors together with fibroblast feeder cells has been widely applied in the area of cancer research including establishment of individual patient originated cancer models and individualized treatment plans (Liu et al. 2012, 2020; Wu et al. 2020). Indeed, conditional reprogramming technology has been recognized as one of the key new technologies by National Cancer Institute precision oncology and included in human cancer model initiatives program (https://ocg.cancer.gov/programs/hcmi/research). Conditional reprogramming allows for the enrichment of cancer cells from urine (for bladder cancer), blood (for prostate cancer), pleural effusion (for non-small cell lung carcinoma), and from small biopsies and cryopreserved specimens of solid tumor tissues. This helps various biological assays including chemosensitivity testing for rapid screening of candidate drugs and developing individualized treatment plans (de Sousa et al. 2020; Liu et al. 2020; Palechor-Ceron et al. 2019; Wu et al. 2020). For example, conditional reprogramming helped to identify therapeutic strategies for a patient with recurrent respiratory papillomatosis with chemoresistant and progressive disease (Yuan et al. 2012), perform personalized drug sensitivity screening for tongue squamous cell carcinoma using patient-derived matched normal and tumor models (Palechor-Ceron et al. 2019) and for bladder cancer (Kettunen et al. 2019), and to identify novel effective drug combination therapeutic strategies for prostate cancer (Vondalova Blanarova et al. 2017) and non-small cell lung cancer (Crystal et al. 2014).

## Future Directions

The ROCK activity contributes to versatile functions on the cell biology through its impacting on cytoskeletal-associated proteins. Accumulating evidence has shown that ROCK signaling has a vital role in normal embryonic development, a period when the biological functions of ROCK1 and ROCK2 isoforms appear to be largely redundant. There is rapidly increasing interest in employing ROCK inhibitors in stem cell research. Supplementing cell culture with the first generation of ROCK inhibitors including Y27632 and fasudil has already been included in most protocols. This approach has been proved to be simple and efficient in expanding stem cells from normal, tumor and other pathological origins, maintaining their pluripotency, and facilitating their differentiation into desired cell types. This approach provides a platform for a wide spectrum of applications including disease modeling, drug evaluation, drug discovery, regenerative medicine and precision medicine.

There is also increased agreement that ROCK1 and ROCK2 have non-redundant functions, and pan-inhibition can elicit undesirable biological effects. Most of currently available ROCK inhibitors being used in investigating the role of ROCK in development and stem cell research are still non-isoform selective. In recent years, nanoparticle carrier delivery therapeutics to target tissue or cells, which can reduce off-target effects of ROCK pan-inhibitors, have provided a promising outcome, especially in preclinical studies (Federico et al. 2020; Mietzner et al. 2020). Additionally, ROCK1 and ROCK2 expression and/or activity can be separately regulated by numerous factors that either positively or negatively modify ROCK catalytic activity and/or subcellular localization. A great effort is underway to elucidate inimitable roles of each ROCK isoform in various developmental stages. Regarding the redundant functions, we observed through conditional knockout approach that the requirement for total ROCK activity during embryonic development is cell type dependent (Tables 2 and 3). The approach provides an exclusive way to analyze in detail the requirement for total ROCK isoform gene copy number in different cell types and in various developmental contexts. We anticipate that the innovative delivery strategies together with advanced knowledge of cell type-specific roles of ROCKs will help to bring most desirable therapeutic applications for high-precision correction of specific cell type-related dysfunction/disease. Furthermore, the CRISPR gene editing technology offers a novel approach to analyze in detail the temporal and spatial roles of ROCK isoforms in early embryonic morphogenesis and lineage commitment. The applications of other cutting-edge technologies such as high-resolution tissue imaging, global transcriptomics (Anderson et al. 2018; Misek et al. 2020) and single cell omics will further enrich our knowledge on ROCK signaling in biological systems and human diseases. Through detailed analyses of ROCK-regulated gene expression patterns at global and cellular levels in physiological and pathological status, these advanced approaches will uncover significant biomarkers or crucial molecules in ROCK-mediated pathogenesis. We look forward to witnessing ROCK inhibition as a new therapeutic choice in future medicine.

## Data Availability

All data and materials as well as software application support this article and comply with field standards.

## Abbreviations

ESCs: Embryonic stem cells
EOB: Eyelids open at birth
hESCs: Human embryonic stem cells
hiPSCs: Human induced pluripotent stem cells
KD: Kinase-dead
LIMK: LIM-kinases
miRNA: MicroRNAs
MEFs: Mouse embryonic fibroblasts
mESCs: Mouse embryonic stem cells
MHC: α-Myosin heavy chain
MSCs: Mesenchymal stem cells
MYPT1: Myosin binding subunit of myosin light chain phosphatase
MLC: Myosin light chain
Peri: Periostin
PH: Pleckstrin-homology
RBD: Rho-binding domain
siRNA: Short interfering RNA
SHED: Stem cells from human exfoliated deciduous teeth
UTR: Untranslated regions
UDSC: Urine-derived stem cells
